# Loss of RBMS1 as a regulatory target of miR-106b influences cell growth, gap closing and colony forming in prostate carcinoma

**DOI:** 10.1038/s41598-020-75083-9

**Published:** 2020-10-22

**Authors:** Jaroslaw Thomas Dankert, Marc Wiesehöfer, Sven Wach, Elena Dilâra Czyrnik, Gunther Wennemuth

**Affiliations:** 1 Department of Anatomy, University Hospital Essen, Hufelandstr. 55, 45147 Essen, Germany; 2 Institute of Molecular Urology, University Hospital Erlangen, Erlangen, Germany

**Keywords:** Prostate cancer, Cancer genomics

## Abstract

Prostate carcinoma (PCa) is the second most commonly diagnosed cancer in males worldwide. Among hereditary genetic mutations and nutrient factors, a link between the deregulation of microRNA (miRNA) expression and the development of prostate carcinoma is assumed. MiRNAs are small non-coding RNAs which post-transcriptionally regulate gene expression and which are involved in tumour development and progression as oncogenes or tumour suppressors. Although many genes could be confirmed as targets for deregulated miRNAs, the impact of differentially expressed miRNA and their regulatory target genes on prostate tumour development and progression are not fully understood yet. We could validate RBMS1, a barely described RNA-binding protein, as a new target gene for oncogenic miR-106b, which was identified as an induced miRNA in PCa. Further analysis revealed a loss of RBMS1 expression in prostate tumours compared to corresponding normal tissue. Overexpression of RBMS1 in DU145 and LNCaP prostate cancer cells resulted in diminished cell proliferation, colony forming ability as well as in retarded gap closing. Our results demonstrate for the first time a miR-106b dependent downregulation of RBMS1 in prostate carcinoma. Additionally, we show new tumour suppressive properties of RBMS1 whose observed loss may further elucidate the development of PCa.

## Introduction

Prostate cancer (PCa) is the second most frequently diagnosed non-skin cancer in men worldwide and remains a major health problem in the western world^1,2^. It is a multifocal disease since at diagnosis primary tumours contain multiple and genetically distinct foci of disease^3^. Beneath standard treatments of this cancer including surgery and radiotherapy, patients with advanced PCa non-suitable for radiotherapy or surgery are treated with androgen deprivation or anti-androgen therapy. This treatment effectively controls androgen-dependent tumours but eventually leads to recurrent androgen-independent prostate cancer with frequent metastases^4^. Although androgens and the androgen receptor (AR) as target are one critical factor for the development and progression of prostate tumours, this tumour entity is highly variable in its response to therapies and is clinically heterogeneous^5^. It is therefore very important to define the genetic factors that may contribute to the initial malignancy of prostate cancer. The deregulation of microRNAs (miRNAs) has been recently described as one mechanism that contributes to the induction and growth of various tumours, including prostate cancer. MiRNAs are short non-coding RNA molecules that function as major players of posttranscriptional gene regulation. They preferentially interact with specific sequences in the 3′-untranslated region (3′UTR) of mRNA targets but they may also bind to the 5′UTR or the open reading frame (ORF) of their targets^6,7^. This interaction facilitated by the RISC (RNA-induced silencing) complex containing Argonaute (Ago-) proteins results in an inhibition of protein synthesis, either by translational repression or degradation of the corresponding target mRNA. MiRNAs are described as multivalent, with one miRNA able to target multiple genes whereas one target gene can be regulated by different miRNAs. MiRNAs can function both as tumour suppressors and as oncogenes depending on their controlled target gene in the appropriate tissue. The altered expression of miRNAs in prostate cancer has been studied extensively^8^. We had previously established miRNA expression profiles of prostate cancer at different stages of malignancy by deep sequencing and microarray analysis and identified novel targets for deregulated miRNAs in prostate cancer^9–12^. One of the mostly induced miRNAs was miR-106b which is now well characterized and mainly described as an oncogenic miRNA overexpressed in different cancer types including HCC^13^, gastric^14^, cervical^15^ and renal carcinoma^16^. Although a continual stream of miRNA targets is being reported, the majority of mRNAs regulated by miRNAs remain unknown. In an ongoing effort to identify target genes of miR-106b, we followed a bioinformatic approach using target lists, which were generated by in silico prediction tool TargetScan. We demonstrate RBMS1 as a new regulatory target of miR-106b and confirm the downregulation of RBMS1 in PCa tissue. Furthermore, we investigated RBMS1 function in DU145 and LNCaP prostate cancer cells after overexpression or reduction of RBMS1 and show inhibitory effects of RBMS1 on cell growth, gap closing and colony formation in PCa cell lines.

## Results

### RBMS1 is repressed in prostate tumours and PCa cell lines

**Figure 1:**
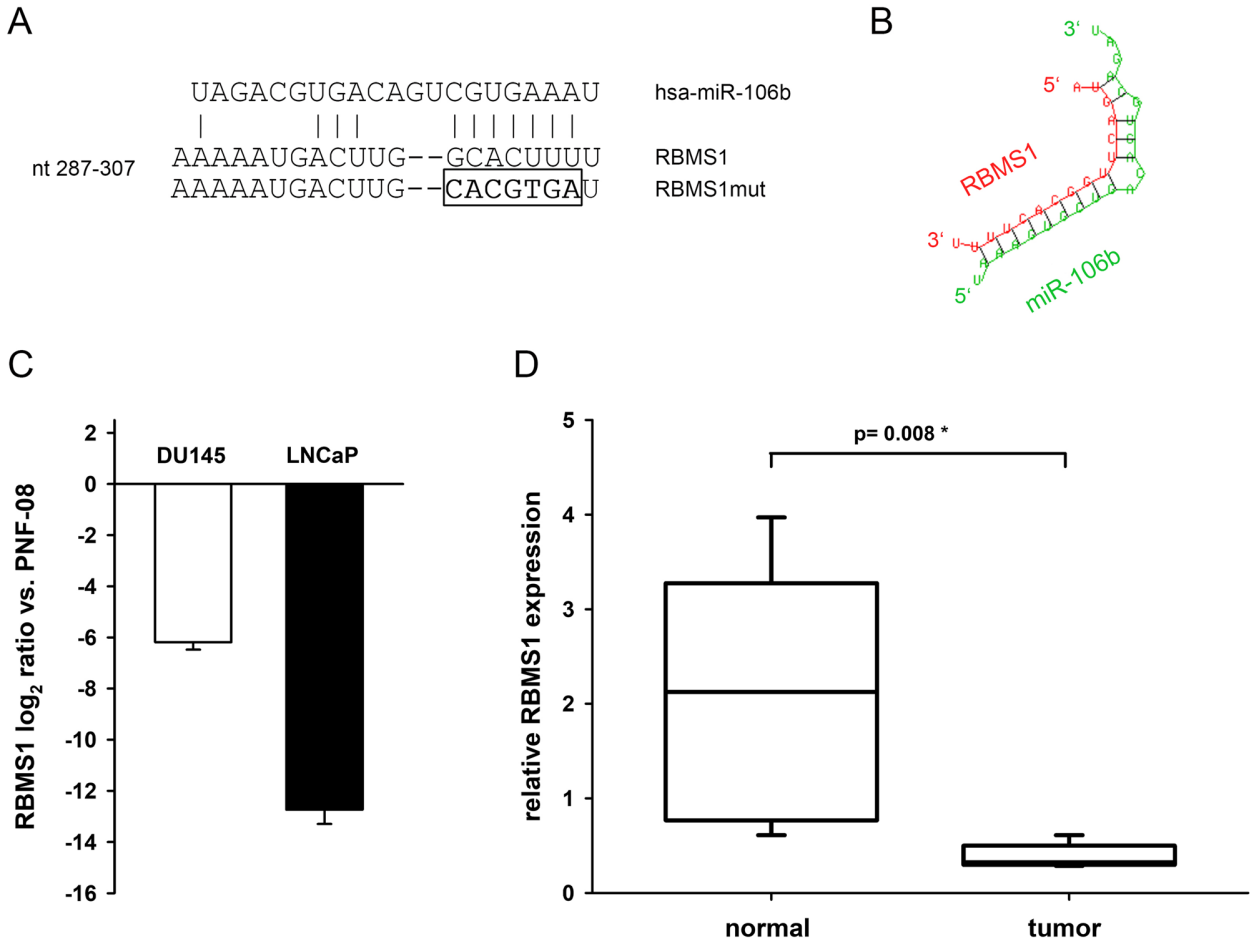
RBMS1 as putative target for miR-106b is downregulated in PCa cell lines and primary prostate tumour tissue. (**A**) The predicted binding site for miR-106b inside the RBMS1 3′UTR including the mutation site of its seed sequence and (**B**) the secondary structure of the miR-106b-RBMS1 hybridization created using RNAhybrid online tool (https://bibiserv.cebitec.uni-bielefeld.de/rnahybrid). (**C**) Expression levels of RBMS1 in DU145 and LNCaP cells relative to primary PNF-08 cells. Total mRNA was extracted and the relative levels of RBMS1 was determined by qRT-PCR. Levels were quantified relative to the amounts observed in PNF-08 cells. (**D**) In total RNA, extracted from 5 pairs of primary CaP (tumor) and corresponding non-tumor (normal) prostate tissues, the relative expression of RBMS1 was determined by qRT-PCR. The data is shown as mean ± SEM performed in triplicates.

We previously described miRNA profiles of PCa by deep sequencing and microarray analysis and detected an induction of miR-106b^10,12^. These findings had meanwhile been confirmed by several groups^17,18^. We performed a bioinformatic analysis to predict further target genes for miR-106b by TargetScan revealing *RBMS1* as a putative target. The 3′UTR region of the RBMS1 mRNA, including the predicted miR-106b interaction site, and the secondary structure of the miRNA-target hybridization is schematically shown in Figs. 1A,B. To examine *RBMS1* expression in PCa, we initially investigated expression levels of RBMS1 mRNA in PCa cell lines and PNF-08 cells by qRT-PCR and we found a reduced expression of RBMS1 transcript in DU145 and LNCaP cells compared to PNF-08 (Fig. 1C). Furthermore, we analysed primary PCa tissue samples and detected a matchable strong reduction of RBMS1 mRNA in tumours compared to the corresponding healthy prostate tissue (Fig. 1D).

### miRNA-106b interacts with the RBMS1 3′UTR and inhibits protein expression in PCa cell lines

**Figure 2:**
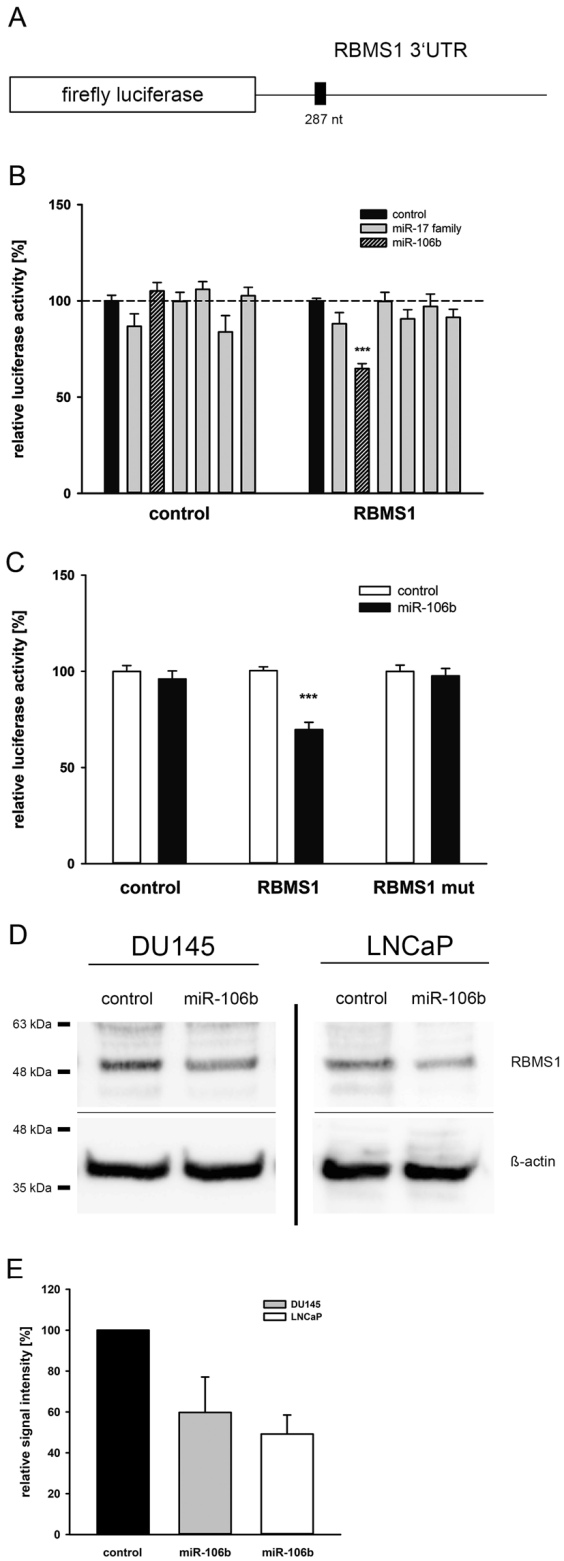
Response of RBMS1 3′UTR and protein expression towards miR-106b. (**A**) The RBMS1 3′UTR fragment was cloned behind the luciferase reporter gene of the pMIR vector. (**B**) The reporter gene construct was expressed with the miRNA expression constructs of the miR-17 family or with the empty pSG5 vector as control in the indicated combinations. Results represent the mean of at least four independent experiments performed in duplicates. The dashed line represents the luciferase activity of the empty luciferase reporter plasmid with the empty pSG5 vector which was set to 100% (***p < 0.001). (**C**) Reporter gene vector containing mutated miR-106b binding site in the RBMS1 3′UTR was co-expressed with miR-106b expression plasmid. Luciferase activity of the reporter vector without miRNA expression was set to 100% (***p < 0.001). (**D**) DU145 or LNCaP cells were transfected either with control vector or miR-106b expression vector. 48 h post-transfection, the protein expression of RBMS1 was determined by Western blot using ß-actin as loading control. Representative cropped Western Blots of RBMS1 detection after miRNA overexpression in DU145 and LNCaP cells from four independent experiments. Full-length blot is presented in Supplementary Figure S5. (**E**) For determination of relative RBMS1 downregulation, each four Western Blots of DU145 and LNCaP cells transfected either with control vector or miR-106b expression vector were densitometrically quantified in relation to the corresponding ß-actin band as loading control. The data is shown as mean ± SEM whereas the control lane intensity was set to 1.

After the initial target prediction, we tested the putative regulatory effect of miR-106b on the RBMS1 3′UTR using Luciferase Reporter Gene Assays. As the miR-17 family, containing *miR-106a/b, -17, -20a/b* and *miR-93*, shares the same seed sequence, which is crucial for target mRNA binding, we also analysed the remaining miR-17 family members. A fragment of the RBMS1 3′UTR containing the predicted miRNA binding site was inserted into a luciferase reporter vector as depicted in Fig. 2A, and was co-transfected with the corresponding miRNA expression vectors into HEK293T cells. Only miR-106b significantly reduced the luciferase reporter gene activity under control of RBMS1 3′UTR down to 65% (*p < 0.05) compared to empty reporter gene vector (Fig. 2B). Mutation of the miRNA binding site inside the RBMS1 3′UTR resulted in a total loss of responsiveness towards miR-106b (Fig. 2C). To confirm the regulative capabilities of miR-106b regarding endogenous RBMS1 protein expression, we overexpressed the miRNA in PCa cells and analysed RBMS1 protein expression by Western Blot (Fig. 2D). Ectopic expression of miR-106b significantly reduced RBMS1 protein level by 40% in DU145 and by 50% in LNCaP cells (Fig. 2E). Taken together, our data demonstrates RBMS1 as a new target gene for miR-106b.

### Alteration of RBMS1 expression has an impact on proliferation of PCa cells

**Figure 3:**
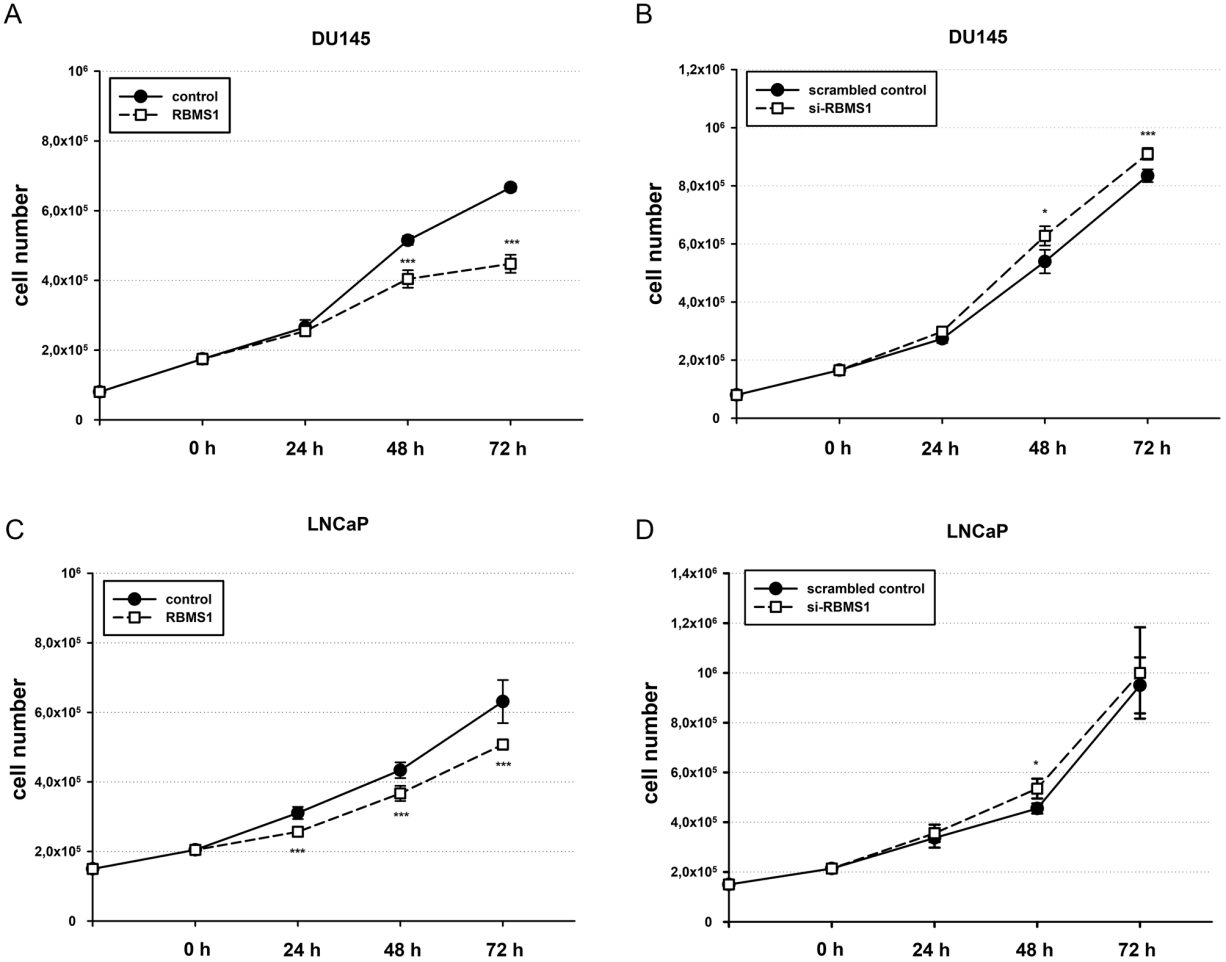
RBMS1 has an inhibiting effect on cell growth of PCa cells. DU145 or LNCaP cells were transfected either with RBMS1 expression vectors or RBMS1 targeting siRNAs and their corresponding controls, respectively, and cells were seeded in a limited cell number. Cell growth was determined by automated cell counting of parallel experiments at 24, 48 and 72 h post-transfection for three independent experiments. The data is shown as mean ± SEM (*p < 0.05; ***p < 0.001).

As the function of *RBMS1* in prostate cells hasn’t been investigated so far, we performed diverse functional analysis of *RBMS1* in DU145 and LNCaP cells. First, both cell lines were transfected with either RBMS1 expression plasmid or siRNAs targeting RBMS1 and cell proliferation was assessed by determing cell numbers over 72 h. Overexpression of RBMS1 led to a reduction of cell number after 48–72 h in DU145 cells compared to control transfected cells (Fig. 3A). In LNCaP cells, the negative effect of RBMS1 on cell growth was already detectable after 24 h but was less distinct compared to DU145 cells (Fig. 3C). On the contrary, siRNA mediated RBMS1 knock-down increased cell numbers in DU145 cells after 24–72 h (Fig. 3B). In LNCaP, cell numbers showed only a slight increase after 48 h (Fig. 3D). We also examined the total number of dead cells throughout the 72 h using the CASY cell counter and we could not detect any changes to be related to a distinct apoptotic effect of *RBMS1* (data not shown). Thus, *RBMS1* seems to inhibit cell proliferation in both investigated PCa cell lines.

### Overexpression of RBMS1 leads to a reduced colony forming ability

**Figure 4:**
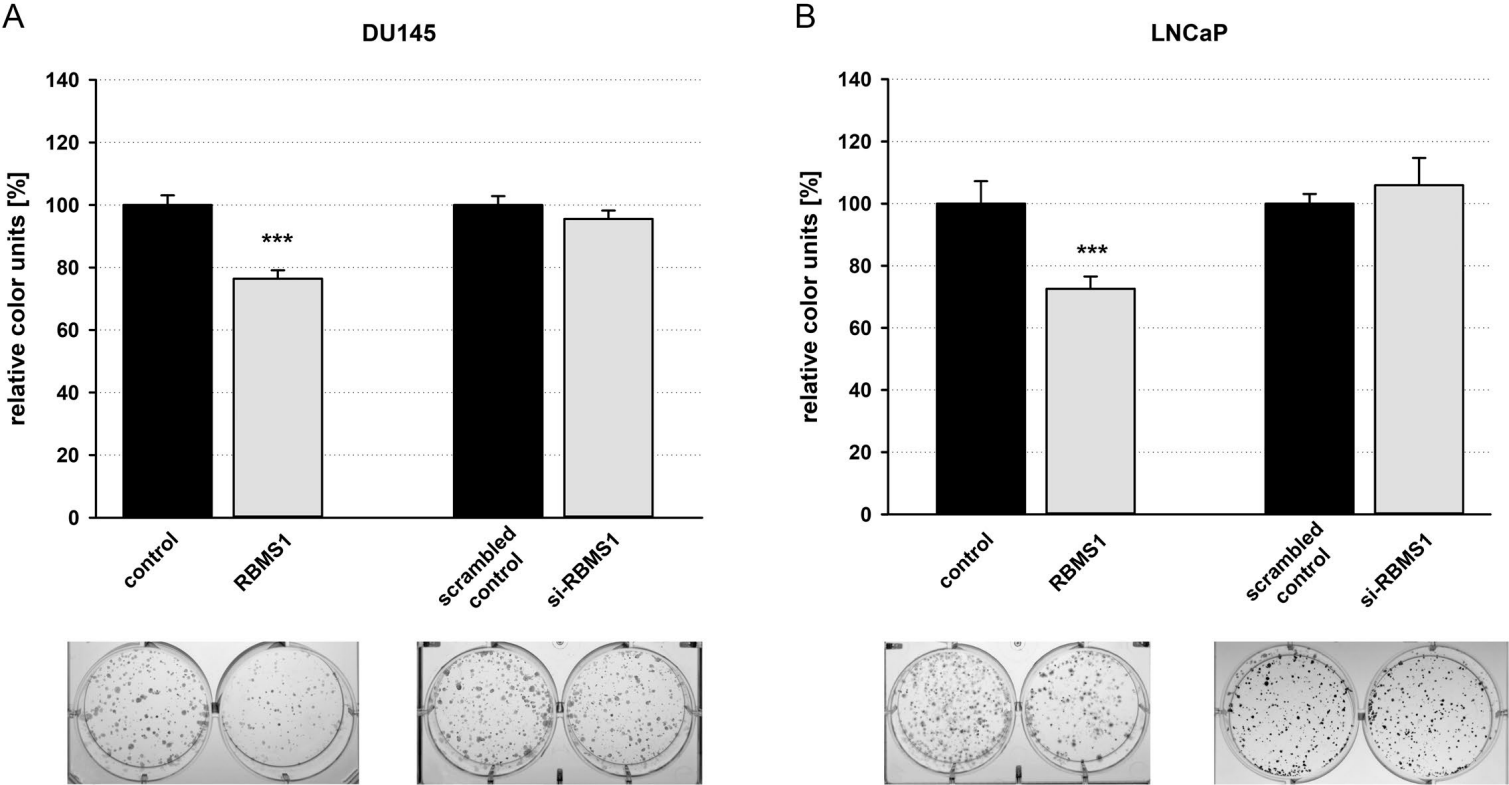
Overexpression of RBMS1 leads to a reduced colony forming ability. DU145 or LNCaP cells were transfected either with RBMS1 expression vectors or RBMS1 targeting siRNAs and their corresponding controls, respectively, and cells were seeded in a limited cell number. 10 days (DU145, (**A**)) or 15 days (LNCaP, (**B**)) after seeding, colonies were stained with crystal violet. Colony formation was quantified by densitometry analyses. Data show the mean and ± SEM of the densitometry analysis of three independent experiments (***, p < 0.001).

Next, we analysed the impact of *RBMS1* on the ability to form colonies. For this, RBMS1 was overexpressed by transfection of expression plasmids or knocked down by using siRNAs in DU145 and LNCaP cells following cell culture of highly diluted cells for 10–15 days. Overexpression of RBMS1 resulted in a diminished colony number by 22% after 10 d in DU145 cells (Fig. 4A) and by 25% after 15 d in LNCaP compared to the corresponding controls, respectively (Fig. 4B). Knock-down of RBMS1 didn’t show any significant influence on the colony forming ability of both cell lines.

### RBMS1 modulates gap closing behaviour of PCa cells

**Figure 5:**
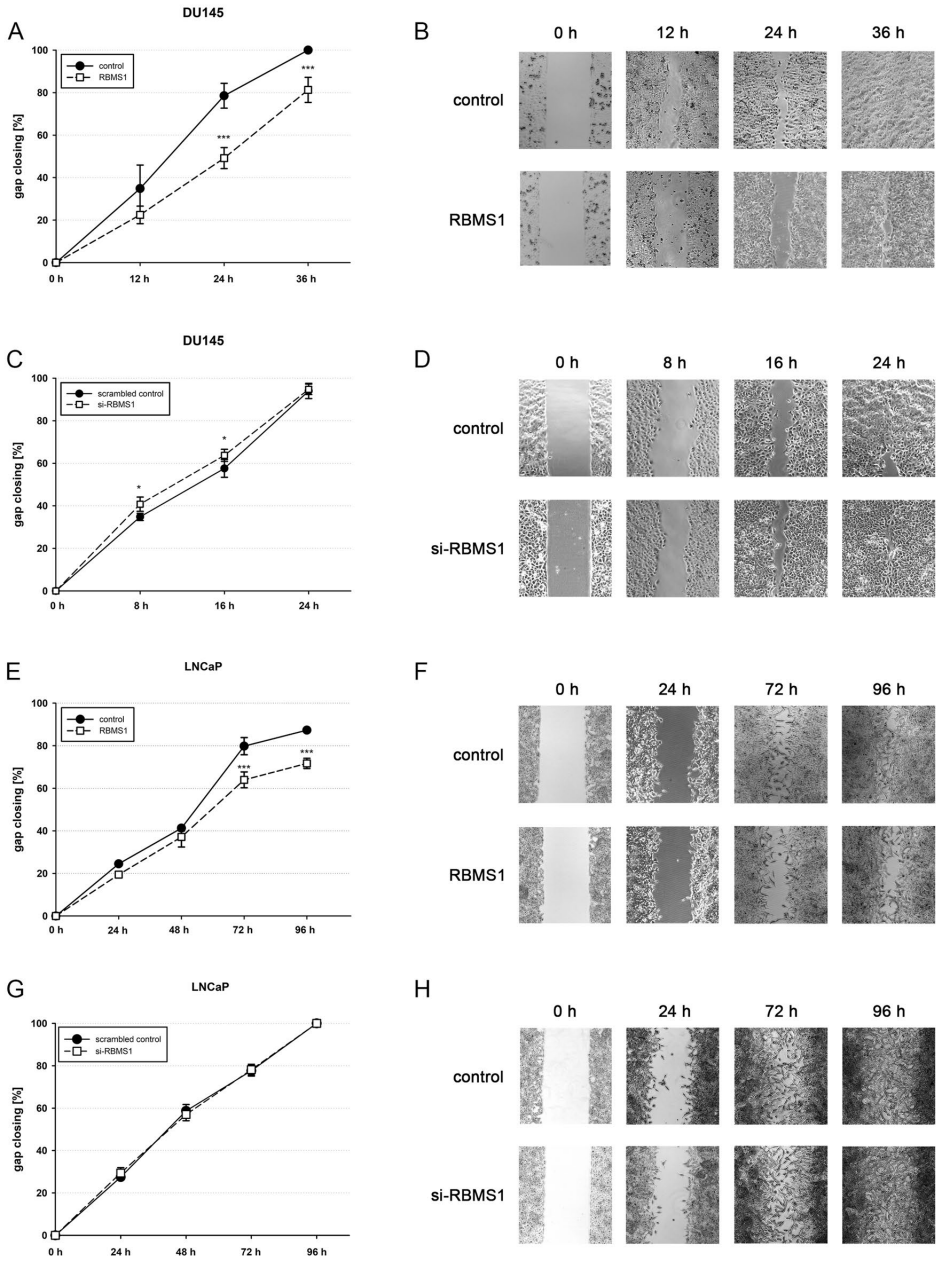
Reduction of gap closing by RBMS1 in PCa cells. DU145 (**A**,**C**) or LNCaP cells (**E**,**G**) were transfected either with RBMS1 expression vectors or RBMS1 targeting siRNAs and their corresponding controls, respectively, and cells were seeded into a ibidi Culture-Insert system to create a defined cell-free gap. Gap closing was monitored for 36 h (DU145) or 96 h (LNCaP) in three independent experiments. For each experiment, the gap width after removing the ibidi culture insert at 0 h was normalized to 0% and three independent visual fields per time point and replicate were examined (*p < 0.05; ***p < 0.001). (**B**, **D**, **F**, **H**) Representive pictures at different time points are depicted.

Finally, we examined gap closing behaviour of PCa cells after manipulation of *RBMS1* expression. After transfection of DU145 and LNCaP cells with either RBMS1 expression plasmid or siRNAs targeting RBMS1 and the corresponding controls, cells were seeded into ibidi chambers including a distinct gap. Once the chamber inlays were removed, cells were observed until gap closing and the area of the remaining gap was related to the initial gap width. DU145 cells showed a diminished gap closing due to RBMS1 overexpression after 24 and 36 h (Fig. 5A,B) whereas cells with lower RBMS1 amounts after knock-down exhibited a slightly induced gap closing behaviour after 8 and 16 h (Fig. 5C,D). In LNCaP cells, RBMS1 induction caused marginally lower gap closing after 72 and 96 h (Fig. 5E,F) though RBMS1 knock-down had no obvious effect (Fig. 5G,H). Our data indicate RBMS1 as a protein with tumour suppressive properties in PCa cell lines which is diminished in primary prostate carcinoma tissue.

## Discussion

We show for the first time a deregulation of *RBMS1* in connection with prostate cancer and demonstrate its decline in primary prostate tumours as well as in the PCa cell lines DU145 and LNCaP by qRT-PCR. LNCaP express AR and PSA, show hormone dependency and are the gold standard of PCa cell lines for in vitro studies whereas DU145 are hormone independent without AR and PSA expression and represent advanced and castration resistant PCa stages. We chose these cells instead of PC3, another frequently used PCa cell line, as studies reported that PC3 show more characteristics of neuroendocrine carcinoma rather than adenocarcinoma^19^. The downregulation of RBMS1 in both, LNCaP and DU145, cell lines indicates a general event in PCa independent of PCa progression state or androgen receptor signalling.

We further identified *RBMS1* as a new target gene for conversely deregulated *miR-106b* in prostate carcinoma. Reporter gene assays showed a direct interaction between miR-106b and the RBMS1 3′UTR inhibiting reporter gene expression, whereas the overexpression of miR-106b in PCa cells reduced endogenous RBMS1 protein levels. MiR-106b is encoded in the *miR-106b*∼*25* cluster additionally containing *miR-25* and *miR-93*. These miRNAs further belong to the *miR-17* family sharing the same seed sequence and show a diverse expression pattern in different tumour entities where they can either promote or inhibit carcinogenesis. Interestingly, only miR-106b bound to RBMS1 3′UTR indicating that a matching seed sequence is not sufficient for translational inhibition of a putative target gene in contrast to other published targets regulated by the entire miR-17 family^20^. The *miR-106b∼25* cluster has, as mentioned above, mainly oncogenic roles in many tumour entities whereas the other *miR-17* family members including the *miR-17–92* cluster are diversely described in its functions^21,22^. In case of prostate cancer, the *miR-106b∼25* locus on chromosome 7 is markedly overexpressed and genetically amplified in prostate cancer^23^ while alterations in the expression profile of *miR-17–92* cluster in PCa cells differ in vivo and in vitro showing further need of studies on the role of *miR-17* cluster in prostate tumorigenesis. Surprisingly, we recently observed tumour suppressive capabilities of miR-106b in LNCaP cells which relate to conflictive results on *miR-17* function^20,24,25^. The fact that overexpression of single miRNAs shows inconsistent results regarding modulation of cellular processes could be explained by a possible cooperation of several miRNAs as tumours usually show a multitude of deregulated miRNAs. Poliseno and colleagues observed that miR-93 and miR-106b, which have mild effects on PTEN individually, cooperate with miR-25 to substantially decrease PTEN abundance. This fine-tuning of PTEN protein is important in tumorigenesis, because even slight variations in tumour suppressor levels can have explicit effects on tumorigenesis and progression^18,26^. Yin and colleagues described miR-383 as a regulator of RBMS1 in murine granulosa cell and oocytes^27^. As this miRNA is located in the frequently deleted chromosomal locus 8p22 and it is frequently downregulated in PCa, this possible regulation of RBMS1 in PCa is rather unlikely^28^. In addition to RNA interference by miR-106b as possible cause for the downregulation of RBMS1, Hubberten and colleagues could recently show an interaction between RBMS1 protein plus mRNA and lncRNA CDKN2B-AS1^29^ whereat overexpression of this lncRNA resulted in an reduced RBMS1 expression. CDKN2B-AS1, also known as *ANRIL*, is induced in prostate tumour tissue and could be another explanation for low RBMS1 abundance in PCa^30^. *RBMS1* (RNA binding motif, single stranded interacting protein 1) has been identified in the 1990s as a nuclear single-stranded DNA binding protein that interacts with the enhancer element of the proto-oncogene *c-myc* inducing DNA transcription as well as with c-myc protein promoting cell transformation^31,32^ and is also suggested to regulate DNA replication^33^.

*c-Myc* is a main regulator for cell proliferation and transformation, and its activity underlies numerous cancers^34^. Overexpression of c-Myc can result in the transformation of primary human prostate epithelial cells *in vitro*^35^. Furthermore, c-Myc cooperates with loss of the phosphatase PTEN to induce prostate cancer progression^36^. The expression of *c-Myc* is associated with prostate cancer recurrence and poor prognosis^37^. c-Myc transcripts and proteins are reported to be upregulated in PCa tissues compared to normal prostate tissue. Due to the downregulation of RBMS1 in PCa, other possible mechanisms proposed to promote c-Myc upregulation include gene amplification^38^, regulation by the long-range enhancers^39^ and different transcriptional upregulation^40^. Fan and colleagues further observed that JMJD1A stabilizes c-Myc protein and also increases *c-Myc* transcription through AR-dependent transcriptional activation.

Beside the downregulation of RBMS1 in PCa, the main observation in this study is the impact of RBMS1 on cell growth, colony forming ability and gap closing behaviour in PCa cell lines.

Overexpression of RBMS1 led to reduced cell proliferation, diminished colony formation ability as well as slower gap closing in DU145 and LNCaP cells. Interestingly, knock-down of RBMS1 caused the contrary effect only on cell proliferation and gap closing in DU145 cells whereas LNCaPs showed only a tendency of increased cell proliferation after RBMS1 knock-down. It’s likely that the absent response towards RBMS1 knock-down in LNCaP cells results from the very low endogenous RBMS1 levels in this cell line compared to DU145 (Fig. 1C, S3). Furthermore, regulation of colony forming may be RBMS1 dose dependent, only altered by high RBMS1 level differences after its overexpression. Until now, the function of RBMS1 has only been investigated in HeLa cells and metastatic colon cancer. Iida and colleagues showed that RBMS1 induces apoptosis in a dose-dependent manner as in the control experiments with c-Myc^41,42^. These results support our observation towards tumour suppressive capabilities of RBMS1. The consequence of RBMS1 downregulation in PCa regarding to downstream target gene expression needs further investigation. Leppek and Stoecklin showed that RBMS1 is also able to bind AU-rich elements (AREs) inside mRNAs which direct mRNA degradation^43^. Thus, loss of RBMS1 could cause an increase of transcripts which may be important for tumour formation and progression as AREs are often found in the 3′UTR of mRNAs coding for proto-oncogenes, nuclear transcription factors and cytokines^44^.

In a recent study, we performed microarray analysis of neuroendocrine transdifferentiated LNCaP cells. Interestingly, RBMS1 transcripts were elevated compared to non-treated LNCaPs (Supplementary Fig. S6). Since we detected impaired cell growth, gap closing and colony forming after RBMS1 overexpression in LNCaP cells, these observations confirm the characteristics of NE-like PCa tumour cells, which show a diminished cell proliferation. Additionally, miR-106b was repressed in NE-like LNCaP cells supporting our findings of RBMS1 regulation by this miRNA. Further studies are needed to elucidate the downstream targets of RBMS1, such as genes or transcripts, which are consequently deregulated after loss of RBMS1 in PCa.

In summary, we demonstrate the downregulation of RBMS1 in prostate cancer tissue which may be caused by increased miR-106b expression. MiR-106b directly regulates endogenous RBMS1 expression in PCa cell lines. Furthermore, we show for the first time tumour suppressive properties of RBMS1 in LNCaP and DU145 prostate cancer cells inhibiting cell growth, gap closing and colony forming ability and identify RBMS1 as a new player in prostate carcinoma.

## Materials and methods

### Clinical samples

Matched tissue samples from prostate cancer and adjacent non-cancerous tissue were prepared from prostatectomy specimens from men with so far untreated prostate cancer between 2008 and 2010. All patients gave informed consent. The study is based on the approval of the Ethics Committee of the University Hospital Erlangen (No. 3755, dated Feb. 2008). All methods were performed in accordance with the relevant guidelines and regulations.

### Cell lines

Cell line description and cultivation has already been described elsewhere^10,19^. The human embryonic kidney 293 cell line containing the SV40 T-antigen (HEK293T; RRID:CVCL_0063) and the human prostate cancer cell lines LNCaP (RRID:CVCL_0395) and DU145 (RRID:CVCL_0105) were purchased from the American Type Culture Collection (ATCC/LGC Standards GmbH, Wesel, Germany). Primary human normal prostate fibroblasts (PNF-08) were kindly provided by Prof. Gerhard Unteregger (Dept. of Urology, University of Saarland Medical School). The number of passages between thawing and use in the described experiments was < 10. HEK293T and DU145 cells were cultivated in DMEM (Thermo Scientific, Schwerte, Germany) supplemented with 10% heat-inactivated FCS (Sigma Aldrich, Hamburg, Germany), Penicillin (100 U/ml) and Streptomycin (100 µg/ml). LNCaP cells were grown in RPMI 1640 (Thermo Scientific, Schwerte, Germany) supplemented with 10% heat-inactivated FCS, l-Glutamin (1 mM final concentration), Penicillin (100 U/ml), Streptomycin (100 µg/ml) and sodium pyruvate (1 mM final concentration). For Mycoplasma testing, cells cultured on coverslips in 12-well plates were fixed with methanol for 15 min, mounted on slides with VECTASHIELD mounting medium containing DAPI (Vector Laboratories, Burlingame, CA, USA) and examined with a fluorescence microscope (Nikon Eclipse Ts2, Tokia, Japan). All experiments were performed with mycoplasma-free cells. Cell lines used were frequently examined regarding morphology, doubling time and growth. All cell lines have been authenticated using STR profiling within the last year.

### RNA extraction and quantitative real-time PCR

QRT-PCR description for cell lines has already been described elsewhere^19^. Total RNA extraction from cell lines and tissue specimen was performed using peqGOLD RNAPure (Peqlab, Erlangen, Germany) according to the manufacturer's instructions. For mRNA analysis in cell lines, cDNA synthesis was performed with the High Capacity cDNA Reverse Transcription Kit (Applied Biosystems, Darmstadt, Germany) using 1 µg of total RNA and RT- random primers. QRT-PCRs were performed with the iQ5 real-time PCR detection system (BioRad, Munich, Germany) using sequence-specific primers and VeriQuest SYBR Green qPCR Master Mix (Affymetrix, Schwerte, Germany) according to the manufacturer’s protocols. All PCRs were measured in duplicates in a final volume of 25 µl containing 50 ng cDNA. The thermal cycling conditions were as follows: 95 °C for 10 min followed by 45 cycles of 95 °C for 15 s, 58 °C for 30 s and 60 °C for 45 s. For quality control, melting curve analysis was performed. Calculation of relative mRNA expression was carried out using the ΔΔCt method with 18S rRNA as endogenous reference. The qRT-PCR primers for detection of RBMS1 expression in cell lines are listed in Supplementary Table S1. To quantify the mRNA levels in tumour specimen, 1 μg of total RNA was reverse transcribed using the Dynamo cDNA synthesis system (Thermo Scientific, Darmstadt, Germany) according to the manufacturer’s recommendations. Quantitative PCR was performed in a StepOne plus real-time PCR system (Thermo Scientific, Darmstadt, Germany) in a final volume of 10 μl containing 1 × gene expression master mix, 1 × gene specific primers and detection probe and cDNA corresponding to 25 ng total RNA. All of the reactions were performed in triplicate. The gene specific primer/probe combinations used were HPRT1 (Hs99999909_m1, Thermo Scientific) and RBMS1 (Hs00249930_s1, Thermo Scientific, Darmstadt, Germany).

### Plasmids and siRNAs

The miRNA expression plasmids were generated by PCR amplification and were described elsewhere^20^. Overexpression of the corresponding miRNA after transfection in HEK293T, LNCaP and DU145 cells was verified by qRT-PCR and is shown in Supplementary Figures S1 and S2. The RBMS1 expression plasmids were generated by PCR amplification of the coding sequence (accession number: NM_016836, nucleotides 445–1665) and inserted into the pSG5 expression vector. Overexpression of RBMS1 after transfection of DU145 and LNCaP cells was verified by Western Blots (Supplementary Fig. S3). The nucleotides 2895–4131 of the RBMS1 mRNA (accession number: NM_016836) containing a part of the corresponding 3′UTR were amplified via PCR using specific primers from human genomic DNA and inserted into pMIR-RNL-TK reporter vector which is described elsewhere^45^. The mutagenesis of the predicted target site seed sequences of pMIR-RNL-TK reporter constructs were performed with QuickChange Site Directed Mutagenesis Kit (Stratagene, Heidelberg, Germany), following the instructions of the manufacturer's manual. The primer sequences used for cloning and site directed mutagenesis are shown in S1 Table. For knock-down of RBMS1, two independent “silencer select” siRNAs (Assay IDs 11865/11866, Thermo Scientific, Schwerte, Germany) and corresponding scrambled control siRNA were used in a concentration of 10 nM and the successful knock-down of RBMS1 after transfection of the corresponding siRNAs was validated by Western Blot (Supplementary Fig. S4). For functional assays, a 1:1 mixture of both siRNAs was used.

### Dual-luciferase assay

Luciferase assay description has already been published elsewhere^11,19^. In brief, 2 × 10^5^ HEK293T were seeded per well in 24-well plates and were cultivated for 24 h. Cells were transfected with 0.8 μg of expression plasmid (pSG5-miR or empty pSG5) and 0.2 µg reporter plasmid (pMIR-RBMS1, pMIR-RBMS1mut, or empty pMIR) using Polyfect Transfection Reagent (Qiagen, Hilden, Germany) and Luciferase Reporter Assays were performed 48 h after transfection using the Dual-Luciferase Reporter Assay System following the instructions of the manufacturer's protocol (Promega, Mannheim, Germany).

### Western blotting

Western blotting description has already been published elsewhere^19^. For transfection of LNCaP and DU145 cells, 6 × 10^5^ cells were cultivated in 6-well plates. After 24 h, cells were transfected with 2 μg of expression plasmid DNA (pSG5-miR-106b, pSG5-RBMS1 or empty pSG5) or 10 nM siRNA (si-RBMS1 or scrambled siRNA) using jetPRIME transfection reagent (Polyplus transfection, Sélestat, France). Cells were lysed 48 h after transfection with 2 × sample buffer (130 mmol/l Tris/HCl, 6% SDS, 10% 3-mercapto-1,2-propandiol, 10% glycerol, and 0.05% bromophenol blue). 30 μg of extracted proteins were separated by 9% Tricine-SDS–Polyacrylamide-Gel electrophoresis and transferred to a nitrocellulose membrane (Whatman, GE Healthcare, Freiburg, Germany) by electroblotting. For immune detection, the primary anti-RBMS1 monoclonal mouse antibody (clone OTI2H1, Thermo Scientific, Schwerte, Germany) and anti-ß-actin monoclonal HRP antibody (clone AC15, Sigma-Aldrich, Hamburg, Germany) were used. Secondary goat anti-mouse HRP clone 31430 antibody was purchased from Pierce (Thermo Scientific, Schwerte, Germany). Bands were visualized by ECL plus Western Blotting Substrate from Pierce (Thermo Scientific, Schwerte, Germany) and the Fujifilm LAS-3000 gel documentation system (Kleve, Germany).

### Colony formation assay

Colony formation assay description has already been published elsewhere^19^. Briefly, 6 × 10^5^ DU145 or LNCaP cells were seeded in 6-well plates and transfected with 2 µg expression plasmid DNA (pSG5-RBMS1 or empty pSG5) or 10 nM siRNA (si-RBMS1 or scrambled siRNA) using jetPRIME (Polyplus transfection, Sélestat, France). 24 h after transfection, cells were detached by trypsin and resuspended in medium, seeded in 6-well plates (2500 cells/well) and cultured for 10–15 additional days. After medium replacement cultures were stained with 0.4% crystal violet, fixed and washed 3 times with PBS. Wells were photographed and densitometrically analysed by ImageJ 1.48v (National Institute of Health, Bethesda, USA).

### Cell proliferation assay

1.6 × 10^5^ LNCaP or 8 × 10^4^ DU145 cells were seeded in 12-well plates, transfected with 1 µg expression plasmid DNA (pSG5-RBMS1 or empty pSG5) or 10 nM siRNA (si-RBMS1 or scrambled siRNA) and cultivated for 24–96 h. For measuring cell numbers on day 0–3 after transfection, cells were detached with trypsin and resuspended in 1 ml medium. Cell numbers were determined by CASY 1 cell counter (Schärfe System, Reutlingen, Germany).

### Gap closing assay

Gap closing assay description has already been published elsewhere^46^. In summary,

2 × 10^5^ DU145 or LNCaP cells were seeded in 12-well plates and transfected with 1 µg expression plasmid DNA (pSG5-RBMS1 or empty pSG5) or 10 nM siRNA (si-RBMS1 or scrambled siRNA) on the following day. After 24 h, cells were detached, resuspended in medium and counted with the automatic cell counter CASY Cell Counter (Roche Innovatis AG, Bielefeld, Germany). After centrifugation for 3 min at 900 rpm, the cell pellet was dissolved in enough medium to generate a concentration of 8 × 10^5^ cells/ml. 70 µl of the cell suspension were applied into each well of the “ibidi culture insert 2 Well” system (Ibidi, Gräfelfing, Germany) positioned in a well of a 6-well plate. On the next day, the culture insert was removed, cells rinsed with medium once and then covered with fresh growth medium. Migrating cells were observed and documented until full gap closing: 24–36 h for DU145 and 96 h for LNCaP cells. For every measurement time point, three pictures were taken, evaluated by ImageJ and averaged.

### Target prediction

MiRNA target prediction was carried out using TargetScan (release 7.1; https://www.targetscan.org/).

### Software and Data analysis

MiRNA-target hybridization was performed using RNAhybrid tool, which is available online for free (built September 18 2017; https://bibiserv.cebitec.uni-bielefeld.de/rnahybrid)^47^.

Statistical evaluation of the luciferase assays, real-time qRT-PCRs, cell proliferation, colony formation and gap closing assays were performed with SigmaPlot 12 (Systat, Erkrath, Germany) using Student’s t-test analysis in case of normal distribution, otherwise by Mann–Whitney test. All statistical tests were performed as two-sided and p-values of < 0.05 were considered as significant. Western blots were quantified by ImageJ 1.48v (National Institute of Health, Bethesda, USA).

## Supplementary information

Supplementary Information

## Data Availability

All data generated or analysed during this study are included in this published article and its Supplementary Information files.
