# Supplementary material for: Loss of RBMS1 as a regulatory target of miR-106b influences cell growth, gap closing and colony forming in prostate carcinoma
Source: Sci Rep. 2020 Oct 22;10:18022. doi: 10.1038/s41598-020-75083-9 (PMC7582885; doi:10.1038/s41598-020-75083-9)
Supplement: Supplementary file 1 — Supplementary Information [file 41598_2020_75083_MOESM1_ESM.pdf]

Supplementary information

Title: Loss of RBMS1 as a regulatory target of miR-106b influences cell growth, gap closing and colony forming in prostate carcinoma

Authors: Jaroslaw Thomas Dankert[1], Marc Wiesehöfer[1], Sven Wach[2], Elena D. Czyrnik[1], Gunther Wennemuth[1*]

Supplementary Table S1: Primer sequences

**qRT-PCR**	
RBMS1-for	5'-ccagtatctgcaagccaagc-3'
RBMS1-rev	5'-tcctgagttgctactgctgct-3'
**cloning primers**	
RBMS1-3‘UTR-for-SacI	5'-cgagctcgcaaaacgattggctactagc-3'
RBMS1-3’UTR-rev-NaeI	5'-cagccggccagttaccactggcaagtctgtg-3'
RBMS1-for-EcoRI	5’-cggaattcatgggcaaagtgtggaaacagcag-3‘
RBMS1-rev-BamHI	5‘-cgggatccttacttattaggttgaaaggtata-3‘
**site directed mutagenesis**	
RBMS1-3‘UTRmut-for	5'-gaaaaataaaaatgacttgCACGTGAtaaaggtaacttcac-3'
RBMS1-3‘UTRmut-rev	5'-gtgaagttacctttaTCACGTGcaagtcatttttatttttc-3'

Supplementary Figure S1

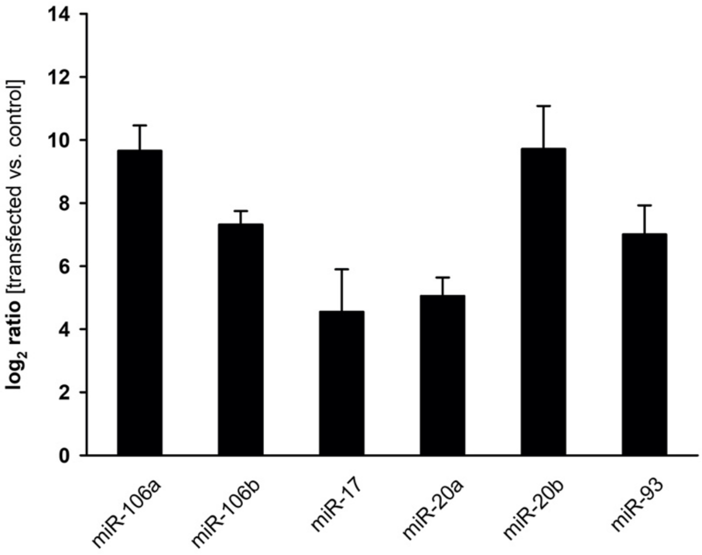

Supplementary Fig. S1: Overexpression of miRNAs after transient transfection of HEK293T cells with expression plasmids for Dual-luciferase assays. HEK293T cells were transfected either with control vector or corresponding miRNA expression vector. 48 h post-transfection, total RNA was isolated and miRNA expression was analyzed by qRT-PCR.

Supplementary Figure S2

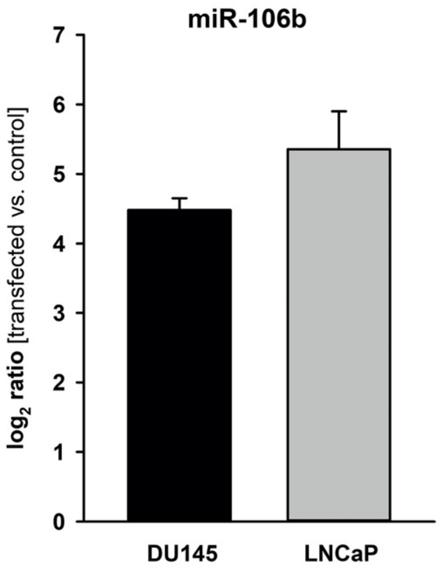

Supplementary Fig. S2: Overexpression of miR-106b after transient transfection of PCa cell lines with miR-106b expression plasmid for Western Blot analysis. DU145 or LNCaP cells were transfected either with control vector or miR-106b expression vector. 48 h post-transfection, total RNA was isolated and miR-106b expression was determined by qRT-PCR.

Supplementary Figure S3

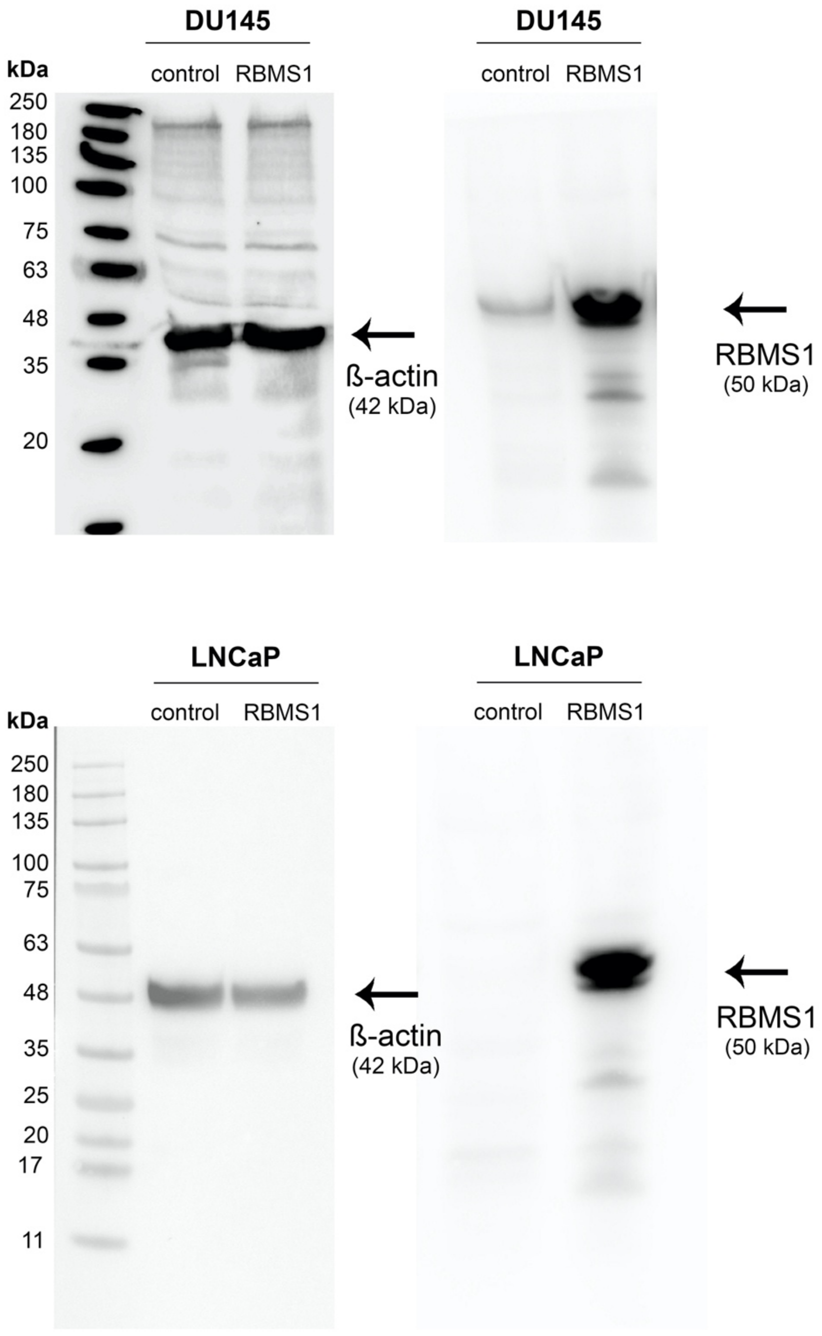

Supplementary Fig. S3: Overexpression of RBMS1 protein after transient transfection of RBMS1 expression plasmid. DU145 or LNCaP cells were transfected either with control vector or RBMS1 expression vector. 48 h post-transfection, total protein was isolated and the protein expression of RBMS1 was determined by Western Blot using ß-actin as loading control.

Supplementary Figure S4

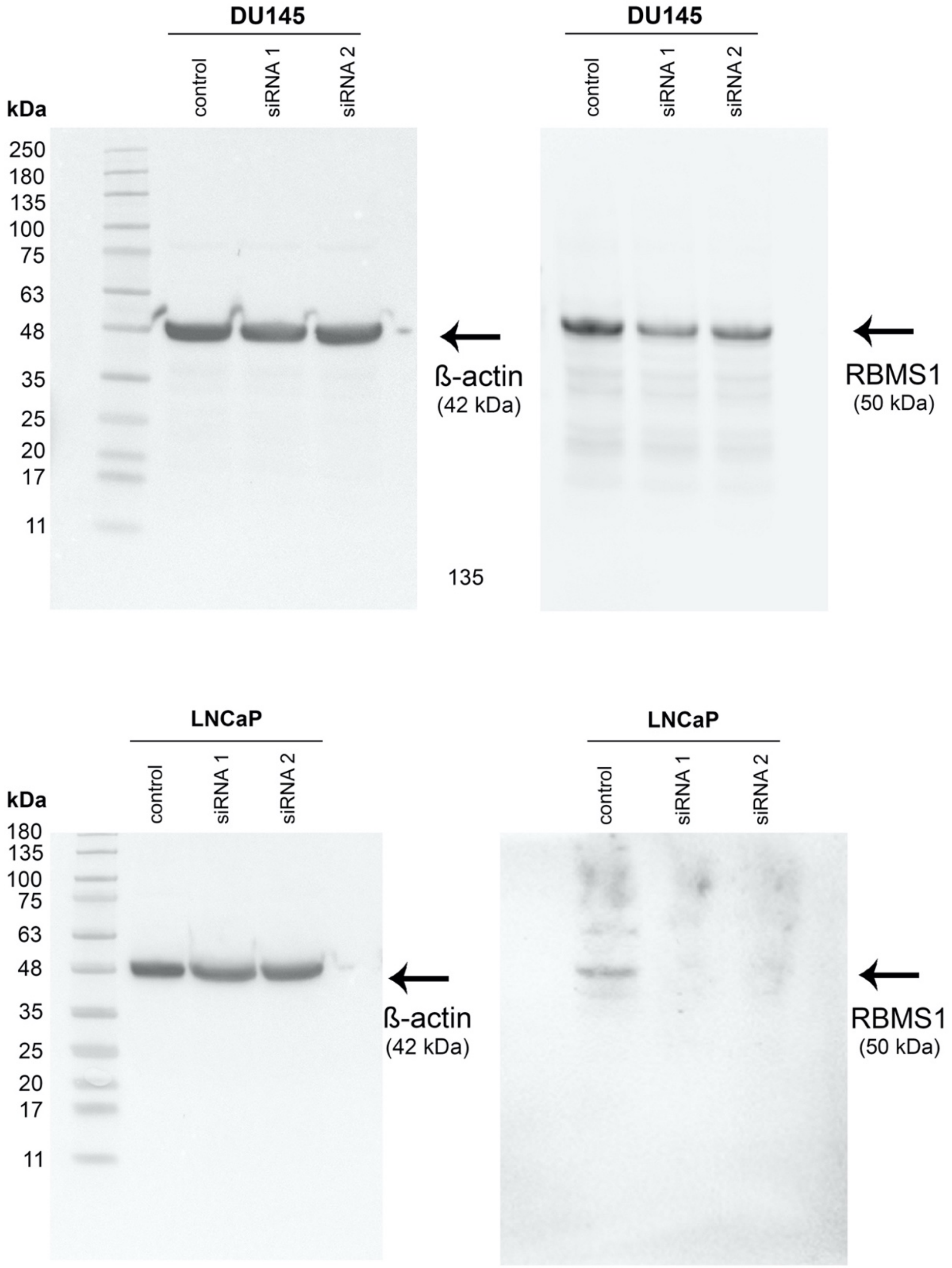

Supplementary Fig. S4: Knock-down of RBMS1 protein after transfection of RBMS1 targeting siRNAs. DU145 or LNCaP cells were transfected either with scrambled control or siRNAs targeting RBMS1 mRNA. 48 h post-transfection, total protein was isolated and the protein expression of RBMS1 was determined by Western Blot using ß-actin as loading control.

Supplementary Figure S5

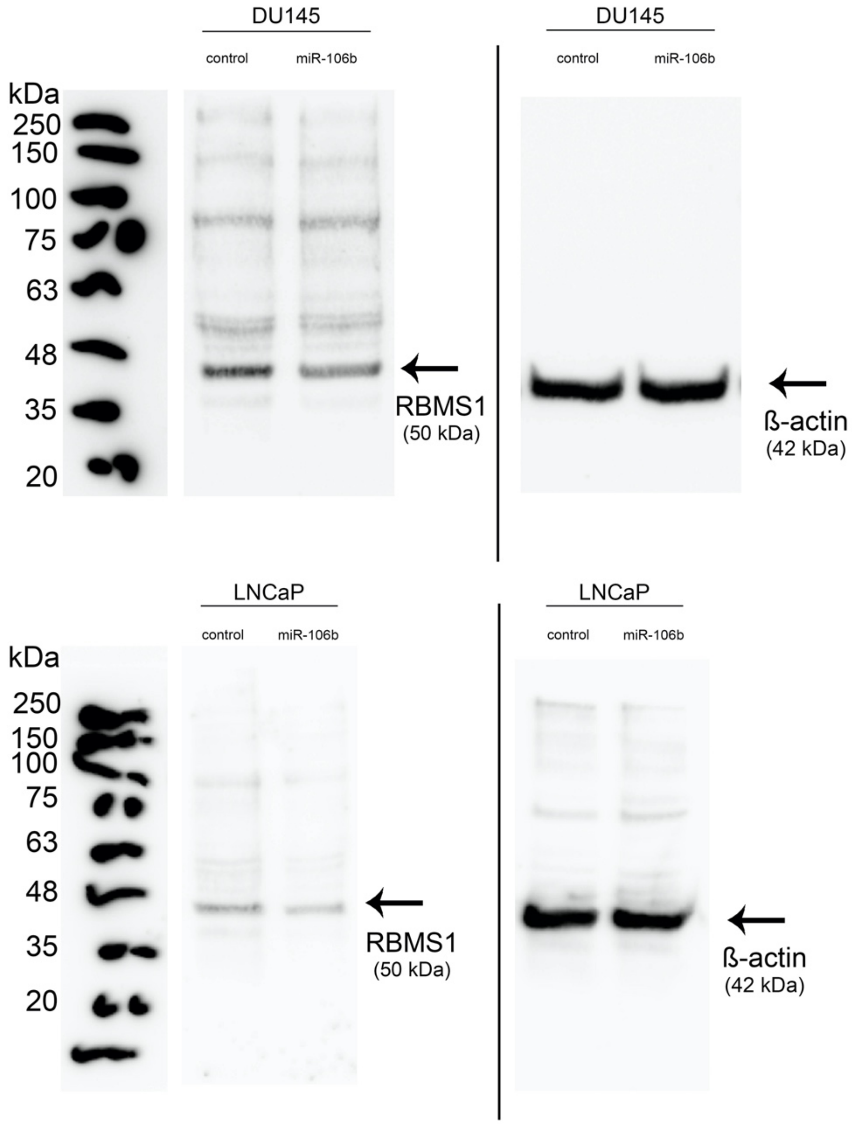

Supplementary Fig. S5: Original RBMS1 and ß-actin blot from Figure 2D.

Supplementary Figure S6

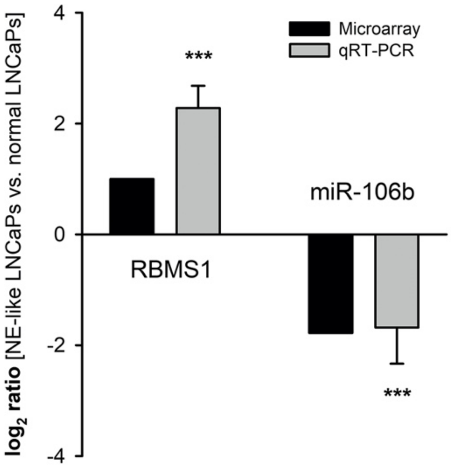

Supplementary Fig. S6: Expression of RBMS1 and miR-106b after neuroendocrine transdifferentiation of LNCaP cells

RBMS1 was predicted to be elevated while miR-106b was predicted to be reduced in NE-transdifferentiated LNCaP as compared to untreated cells in microarray analysis (black bars). The altered expression of RBMS1 and miR-106b was validated by qRT-PCR (grey bars) ***,p<0.001.
